# fNIRS-Guided neuronavigated rTMS augments naming recovery in subacute post-stroke aphasia: a double-blind randomized controlled trial

**DOI:** 10.3389/fnhum.2026.1810169

**Published:** 2026-04-22

**Authors:** Haozheng Li, Dongxiang Fang, Yihao Chen, Shuqi Xu, Ying Wang, Wenwen Wei, Ruofan Zhao, Yixian Lai, Yi Wu, Qing Yang, Ruiping Hu

**Affiliations:** 1 Department of Rehabilitation Medicine & Shanghai Institute of Infectious Disease and Biosecurity, Huashan Hospital, Fudan University, Shanghai, China; 2 National Center for Neurological Disorders, Fudan University, Shanghai, China; 3 National Clinical Research Centre for Aging and Medicine, Fudan University, Shanghai, China; 4 Department of Rehabilitation Sciences, Binzhou Medical University, Binzhou, Shandong, China

**Keywords:** functional near-infrared spectroscopy, individualized target localization, neuroplasticity, repetitive transcranial magnetic stimulation, subacute post-stroke aphasia

## Abstract

**Background:**

Selecting an optimal stimulation target is a persistent barrier to maximizing the efficacy of repetitive transcranial magnetic stimulation (rTMS) for subacute post-stroke aphasia. This trial evaluated an individualized, fNIRS-based targeting strategy derived from task-evoked activation during picture naming.

**Methods:**

We conducted a double-blind, randomized, sham-controlled clinical trial. Patients with first-ever left-hemispheric stroke and subacute aphasia (1–6 months post-onset; 35–80 years) were recruited at Huashan Hospital (2021–2023) and randomized 1:1 to receive fNIRS-guided active rTMS or fNIRS-guided sham stimulation, in addition to 3 weeks of intensive speech-language therapy. Individual targets were defined as the fNIRS channel showing the strongest picture-naming task activation and were delivered under neuronavigation. Active stimulation used 10 Hz, 80% resting motor threshold, 2,000 pulses per session, for 15 sessions (3 weeks); sham used an identical schedule with a sham configuration. Primary/secondary outcomes included the Chinese Boston Naming Test (BNT), WAB-R indices, picture-naming behavioral performance, and task/resting-state fNIRS measures.

**Results:**

Twenty-eight participants were enrolled and 27 completed the protocol (active rTMS: *n* = 14; sham: *n* = 13). Language performance improved over time in both groups; however, active rTMS produced significantly larger gains in confrontation naming on the BNT (Time × Group: *F* = 16.04, *p* < 0.01) and higher picture-naming accuracy (Time × Group: *F* = 20.10, *p* < 0.01), with an additional advantage on the WAB-R naming subscore (Time × Group: *F* = 4.44, *p* < 0.05). Neurophysiologically, task-based fNIRS indicated increased activation in the left dorsolateral prefrontal cortex and left Broca’s area after active treatment, accompanied by strengthened resting-state connectivity between these regions; connectivity change was positively correlated with BNT improvement (*r* = 0.6405, *p* < 0.05).

**Conclusion:**

fNIRS-guided rTMS protocol based on individualized, task-evoked activation mapping is a safe and effective approach for improving picture-naming performance in patients with subacute post-stroke aphasia. The intervention yields promising short-term therapeutic benefits and is associated with enhanced activation in the left dorsolateral prefrontal cortex and Broca’s area, as well as strengthened functional connectivity between these language-related regions.

**Clinical trial registration:**

https://www.chictr.org.cn/showproj.html?proj=61768, Unique identifier: ChiCTR2000038515.

## Introduction

Aphasia is a frequent sequela of stroke, affecting approximately 21–38% of stroke survivors, and it disrupts multiple domains of language processing, including perception, comprehension, and expression (Burton et al., 2023). Consequently, post-stroke aphasia (PSA) is among the most disabling complications after stroke: communication limitations commonly precipitate social withdrawal, diminish health-related quality of life, and impede community reintegration and return to work, while also imposing substantial caregiver burden (Doucet et al., 2012). Relative to stroke survivors without aphasia, patients with PSA experience poorer overall outcomes, longer hospitalizations, and higher healthcare utilization and costs (Ellis et al., 2012; Lazar and Boehme, 2017). Despite conventional interventions such as pharmacotherapy and speech-language therapy, recovery is often incomplete; notably, up to 20% of individuals with PSA do not regain functional communication abilities (Gerstenecker and Lazar, 2019).

Transcranial magnetic stimulation (TMS) is a non-invasive neuromodulation technique that has been increasingly incorporated into neurorehabilitation (Xu and Sun, 2020; Li X. Y. et al., 2024). By generating rapidly changing magnetic fields to induce electric currents in cortical tissue, TMS can modulate cortical excitability and network dynamics (Pell et al., 2011; Chung et al., 2016). In general, low-frequency repetitive TMS (rTMS) and continuous theta-burst stimulation (cTBS) are thought to decrease excitability, whereas high-frequency rTMS and intermittent theta-burst stimulation (iTBS) tend to increase excitability and facilitate neuroplasticity (McDonnell and Stinear, 2017; Bai et al., 2022). In chronic aphasia, stimulation paradigms are typically informed by complementary neuroplasticity models: excitatory stimulation of perilesional regions in the affected hemisphere aims to support recruitment of residual language networks, whereas inhibitory stimulation of the contralesional hemisphere—consistent with the interhemispheric competition framework—seeks to attenuate maladaptive hyperactivity and promote re-engagement of lesioned networks (Dammekens et al., 2014; Hara et al., 2017; Ntasiopoulou et al., 2023).

Although TMS has shown therapeutic promise in chronic aphasia, its application in early-stage aphasia remains comparatively limited (Gholami et al., 2022; Kielar et al., 2022; Wang et al., 2023). Converging evidence from motor recovery suggests a time-sensitive window of heightened neuroplasticity in the early post-stroke period, during which the injured brain may be particularly responsive to neuromodulatory interventions (Hermann and Chopp, 2012; Schevenels et al., 2022). Neuroimaging and stimulation studies further indicate that early post-stroke rTMS can help normalize hemispheric activity, and that successful modulation of network-level dynamics is associated with clinical benefit. Emerging clinical data support this rationale (Du et al., 2019; Chen et al., 2022; Juan et al., 2022), several trials have reported improved language outcomes with early brain stimulation (Zheng et al., 2022; Gan et al., 2024; Dang et al., 2025), and a recent multicenter study across different languages and regions observed that subacute PSA patients receiving rTMS achieved substantially greater gains in naming—approximately double those of control participants—along with effects that appeared more pronounced than typically reported in chronic PSA cohorts (Zumbansen et al., 2022).

However, one of the major unresolved challenges in subacute post-stroke aphasia is how to optimally select stimulation targets. Functional neuroimaging offers a new framework for individualized target localization. In prior work, many investigators have used functional magnetic resonance imaging (fMRI) to identify language task–related targets in patients with post-stroke aphasia (Kakuda et al., 2010; Szaflarski et al., 2011; Ren et al., 2023); nevertheless, several limitations may restrict its applicability in this population. For example, scanner noise may interfere with language processing, and patients with metallic medical implants are often excluded (Irani et al., 2007). Functional near-infrared spectroscopy (fNIRS) represents a well-established and feasible alternative with high concordance to fMRI findings (Chen et al., 2020), while providing superior ecological validity (Butler et al., 2020). Because fNIRS is compatible with facial and mandibular movements, it is particularly suitable for paradigms involving overt speech production—a practical advantage that has also been supported by our prior work (Li H. et al., 2022; Li H. et al., 2024). In addition, fNIRS does not generate a magnetic field and is relatively quiet and comfortable for participants. Chang and colleagues further demonstrated the feasibility of using fNIRS to guide rTMS target selection in a prospective pilot study of chronic post-stroke aphasia (Chang et al., 2022).

Whether such neuroimaging-guided targeting is effective and safe for subacute aphasia remains unclear, despite the premise that subacute patients may exhibit greater neuroplastic potential and heightened responsiveness to rTMS. Naming is among the most critical language functions affected in post-stroke aphasia (Knopman et al., 1984), and TMS has been repeatedly reported to improve naming performance (Gholami et al., 2022). Therefore, the present study aimed to determine, in a double-blind randomized controlled trial, whether individualized target localization based on fNIRS-derived activation during a picture-naming task can yield superior language recovery in post-stroke aphasia. We further used fNIRS to investigate the neuroplastic mechanisms underlying treatment-related changes.

## Materials and methods

### Study design

This clinical trial adopted a double-blind, randomized controlled design. All participants received 3 weeks of intensive speech-language therapy combined with either rTMS or sham rTMS. The study protocol was approved by the Ethics Committee of Huashan Hospital, Fudan University (No. KY2019-512) and was registered in the Chinese Clinical Trial Registry (ChiCTR2000038515). Written informed consent was obtained from all participants, and all procedures were conducted in accordance with the Declaration of Helsinki.

### Participants

From 2021 to 2023, we recruited patients with first-ever left-hemispheric stroke from Huashan Hospital, Fudan University, Shanghai, China. Eligible participants were required to meet the following criteria: (1) age between 35 and 80 years; (2) diagnosis of aphasia confirmed using the Chinese version of the Western Aphasia Battery–Revised (WAB-R) and the Boston Diagnostic Aphasia Examination (BDAE); (3) subacute post-stroke aphasia with time since onset between 1 and 6 months (Burton et al., 2023); (4) native speaker of Mandarin Chinese; (5) right-handed, as assessed by the Edinburgh Handedness Inventory; (6) no history of language disorder prior to stroke; (7) no obvious cognitive impairment; and (8) no contraindications to TMS. Exclusion criteria were: (1) previous symptomatic cerebrovascular events; (2) neurodegenerative or major psychiatric disorders; (3) epilepsy; and (4) hearing or visual impairments that could interfere with trial procedures.

### Randomization and blinding

Participants were randomly assigned in a 1:1 ratio to receive one of two interventions: fNIRS-guided rTMS target stimulation or fNIRS-guided sham stimulation. The randomization sequence was generated by computer, and allocation codes were drawn by participants or their family members. All patients were instructed not to discuss details of the treatment procedures or any stimulation-related sensations with study personnel or other participants. Speech-language therapists, who administered the language assessments, conducted the fNIRS scans, and delivered the intensive language training, were blinded to group allocation. The rTMS operator was blinded to all aphasia assessments, fNIRS results, and therapy plans, and was only responsible for delivering the assigned stimulation protocol.

### Sample size

Based on previous studies of TMS for subacute post-stroke aphasi (Waldowski et al., 2012; Thiel et al., 2013; Gan et al., 2024), we anticipated a moderate effect size of 0.5, with a two-sided *α* of 0.05 and power of 0.80, yielding a minimum required sample size of *n* = 13 per group. Assuming a dropout rate of 10%, we planned to recruit 14 participants for each group.

### fNIRS acquisition

**Figure 1:**
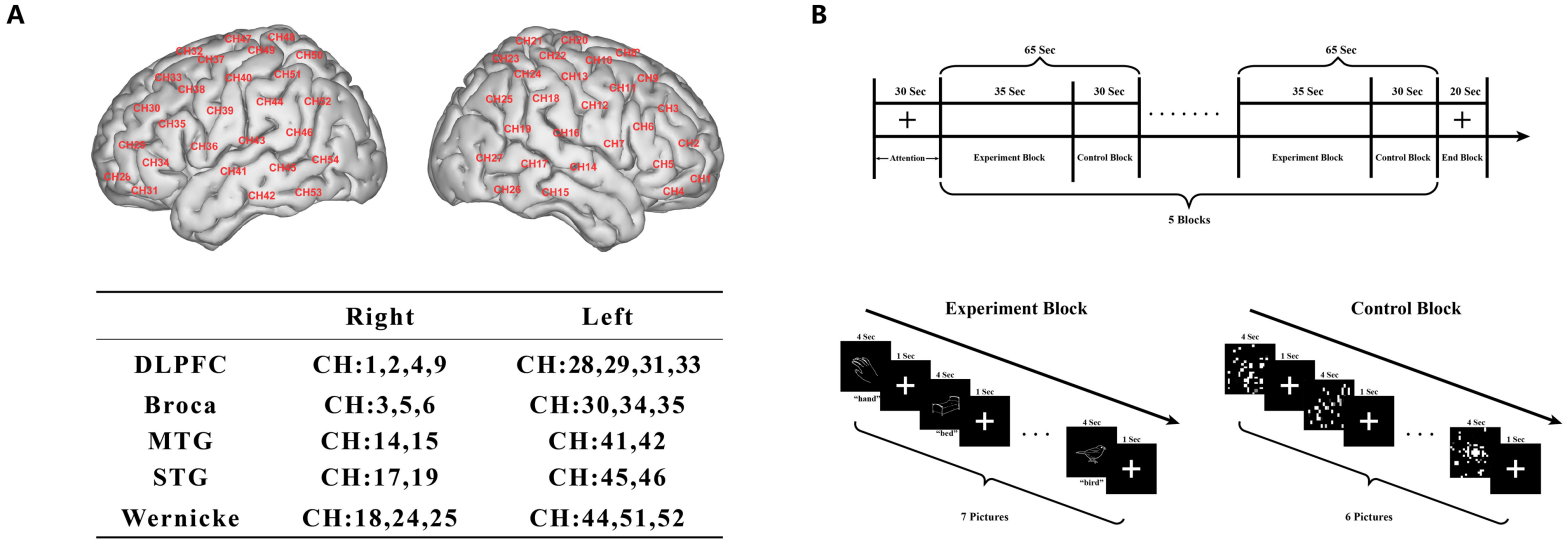
**(A)** fNIRS channel layout on a standard brain template, with 54 channels covering bilateral frontal, temporal, vertex, and occipital regions and grouped into ROIs. **(B)** Block-design picture-naming paradigm comprising an initial fixation period followed by five cycles of experiment blocks and control blocks during fNIRS recording.

A continuous-wave functional near-infrared spectroscopy (fNIRS) system (HuiChuang Medical Company ltd., China) was used to measure changes in oxygenated hemoglobin (HbO) and deoxygenated hemoglobin (HbR) from the scalp and underlying cortex in all participants. The system operated at two wavelengths (730 and 850 nm) with a sampling rate of 11 Hz. The optode montage comprised 22 sources and 16 detectors, forming 54 channels that bilaterally covered the frontal, temporal, vertex, and occipital regions. To obtain Montreal Neurological Institute (MNI) coordinates for each fNIRS channel (defined as the midpoint between each source–detector pair), the spatial positions of all sources, detectors, and anatomical anchor points (Cz, Nz, Iz, AL, and AR) were recorded using an electromagnetic 3D digitizer (FASTRAK, Polhemus, USA). As illustrated in Figure 1A, the cortical projection of each channel was visualized on a standard brain template, and channels were assigned to the following regions of interest (ROIs) based on their mean MNI coordinates: dorsolateral prefrontal cortex (DLPFC), Broca’s area, Wernicke’s area, middle temporal gyrus (MTG), and superior temporal gyrus (STG).

Upon entering the examination room, participants were asked to sit quietly for 3 min, consistent with our previous work (Yang et al., 2025), to allow acclimatization to the experimental environment and the fNIRS setup and to minimize adaptation-related artifacts in the subsequent recordings. After this adaptation period, formal data acquisition commenced. Participants were instructed to close their eyes, refrain from engaging in deliberate or rhythmic mental activities, and remain as still as possible during an 8-min resting-state fNIRS scan. The total duration of the resting-state session was 11 min, including the adaptation period.

Following the resting-state scan, participants completed a picture naming task that has been employed in several of our prior studies (Figure 1B; Li H. et al., 2022; Li H. et al., 2024; Li J. et al., 2024). During a short practice session before the fNIRS task, participants were familiarized with the procedure and example stimuli. The experimental stimulus set consisted of 311 black-and-white line drawings selected from the Yang Yufang picture database (Zhang and Yang, 2003), which had been matched for difficulty and adapted for Chinese cultural relevance. The task followed a block design with alternating task blocks (35 s) and control blocks (30 s), repeated five times, yielding a total task duration of approximately 7–8 min. In each task block, seven object pictures (e.g., a hand) were presented, and participants were instructed to name each picture aloud. In control blocks, scrambled, meaningless figures on a black background were presented to control for low-level visual input. During the picture naming task, vocal responses and reaction times were recorded with millisecond precision using E-Prime 3.0 (Psychology Software Tools Inc., USA) interfaced with a Chronos response box (Psychology Software Tools Inc., USA). A trained research assistant recorded, for each trial, whether the naming response was correct in order to calculate accuracy and to ensure that all participants completed the fNIRS acquisition with adequate task performance.

### Neuroimaging-guided rTMS target localization

**Figure 2:**
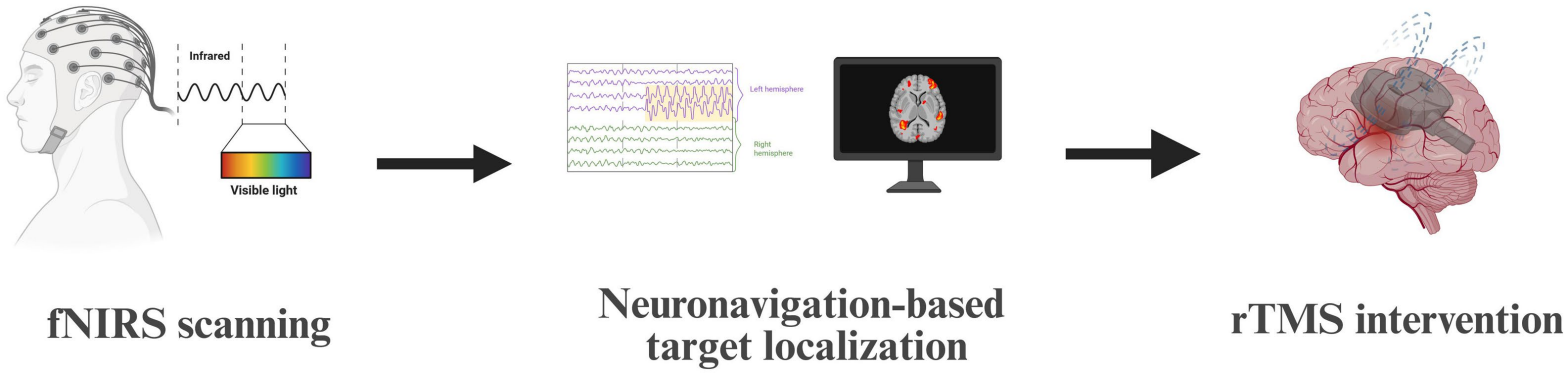
Workflow of fNIRS-guided rTMS targeting.

For each participant, the fNIRS data were analyzed to identify cortical regions showing the strongest task-related activation during picture naming. The channel exhibiting peak activation associated with naming was labeled as the individualized stimulation target (Chang et al., 2022). The intervention was delivered exclusively to the peak activation region in the ipsilesional left hemisphere, as the role of right-hemisphere activation during recovery in the subacute phase remains controversial (Winhuisen et al., 2005; Postman-Caucheteux et al., 2010), these right-hemisphere activation points were not included in the potential stimulation target selection. This functionally defined target was then used to guide neuronavigated rTMS. A frameless neuronavigation system (Visor 2.0, ANT Neuro Inc., the Netherlands) was employed to coregister the fNIRS-derived target with the participant’s scalp and to precisely position the TMS coil over the identified cortical site during stimulation (Figure 2).

### Transcranial magnetic stimulation and speech-language therapy protocol

rTMS was delivered using an MCF-B65 figure-of-eight coil (MagVenture Inc., Denmark). The coil was positioned over the individualized neuroimaging-guided target derived from the fNIRS scan, and stimulation intensity was set at 80% of the resting motor threshold (rMT) of the first dorsal interosseous muscle in the unaffected hemisphere, this intensity was chosen primarily for safety considerations in subacute stroke patients, ensuring effective cortical modulation while minimizing the risk of adverse effects (Ma et al., 2013; Wang et al., 2019). A high-frequency protocol of 10 Hz was applied, with a total of 2,000 pulses per session, delivered in trains of 200 pulses separated by 10-s inter-train intervals (Dammekens et al., 2014). Sham rTMS was administered with the same stimulation parameters, but the coil was rotated so that only its edge touched the scalp, allowing participants to perceive comparable acoustic and vibratory sensations without effective cortical stimulation (Zheng et al., 2022; Gan et al., 2024; Dang et al., 2025). As all patients were naive to TMS, they were unaware of whether they had received real or sham stimulation, a blinding condition that was confirmed by post-intervention self-report. After the intervention, all participants were asked to report which group they believed they had been assigned to (real rTMS or sham stimulation).

Immediately after each rTMS or sham session, all participants received 45 min of intensive speech-language therapy (SLT). Both groups underwent the same impairment-based behavioral therapy, individually tailored to the patients’ language deficits and closely aligned with the intensive SLT protocol used in our previous multicenter randomized controlled trial (Feng et al., 2025). As noted above, speech-language therapists were blinded to group allocation, thereby minimizing potential influences of therapist characteristics or rehabilitation strategies. rTMS (or sham) combined with SLT was administered once daily from Monday to Friday over three consecutive weeks, for a total of 15 treatment sessions.

### Outcome measures

Before the first stimulation session, eligible patients underwent baseline assessment with the Chinese versions of the Boston Naming Test (BNT), the Western Aphasia Battery–Revised (WAB-R), and an fNIRS scan. The BNT and WAB-R were re-administered by the same examiner at the end of the 3-week intervention. At that time, each patient also received a follow-up fNIRS scan. All adverse events occurring during the intervention period and within 1 month after treatment completion were systematically recorded to evaluate the safety of the protocol.

The primary outcome measure was the Chinese version of the BNT (Chen et al., 2014; Li Y. et al., 2022), a validated confrontation naming test consisting of 30 culturally adapted black-and-white line drawings designed to assess picture-naming ability in native Mandarin speakers. The Chinese BNT has been adapted to ensure appropriate word frequency, familiarity, and cultural relevance. Secondary outcome measures included the Chinese version of the WAB-R (Ren et al., 2023; Fang et al., 2025), a standardized clinical language battery for evaluating aphasia severity. The Aphasia Quotient (AQ), derived from the WAB-R, reflects overall severity of language impairment, with a maximum score of 100 and scores below 93.8 indicating aphasia. The WAB-R comprises four core subtests: spontaneous speech, auditory comprehension, repetition, and naming. Additional behavioral indices obtained during the fNIRS picture-naming task included naming accuracy and reaction time (in milliseconds), recorded using E-Prime 3.0 (Psychology Software Tools Inc., USA) interfaced with a Chronos response box (Psychology Software Tools Inc., USA). Stimuli were drawn from the Yang Yufang picture database (Zhang and Yang, 2003), in which items are matched for difficulty and adapted for Chinese cultural relevance. Furthermore, resting-state fNIRS data were used to quantify functional connectivity among language-related cortical regions, reflecting the strength of intra- and inter-regional coupling in patients with aphasia. Our previous work (Li H. et al., 2024) and that of others (Meier et al., 2023; Gan et al., 2024) have demonstrated the specificity and clinical relevance of this connectivity metric. Task-related fNIRS data during picture naming were also analyzed to derive activation amplitudes in language-related cortical areas, providing an index of the magnitude of cortical engagement during naming, which has likewise been shown in our prior studies (Li H. et al., 2022; Li H. et al., 2024) to be informative for characterizing neural reorganization in aphasia.

### Behavioral and fNIRS statistical analyses

Behavioral data were analyzed using SPSS Statistics version 25.0 (IBM Corp., USA). Normality was assessed with the Shapiro–Wilk test, and continuous variables are reported as mean ± standard deviation (SD) when normally distributed. Baseline measures were compared between groups using two-tailed independent-samples t tests for continuous variables and chi-square tests for categorical variables; when distributional assumptions were violated, appropriate nonparametric tests were applied. Pre–post changes in behavioral outcomes were examined using a two-way repeated- measures analysis of variance (RM-ANOVA) with Treatment (active rTMS vs. sham) as the between-subject factor and Time (baseline vs. post-intervention) as the within-subject factor. As a sensitivity analysis, analysis of covariance (ANCOVA) was performed to examine treatment effects on each behavioral outcome, with the post-intervention value as the dependent variable, group (active rTMS vs. sham) as the fixed factor, and the corresponding baseline value, baseline AQ and years of education as covariates. This approach was employed to assess the robustness of the primary findings and to evaluate treatment effects after adjusting for baseline imbalances. Results are presented as adjusted mean differences (AMD) between groups with 95% confidence intervals (CIs) and two-sided *p*-values. Effect sizes are reported as partial *η*^2^. The homogeneity of regression slopes assumption was verified by testing the treatment-by-covariate interaction term prior to the primary analysis. A two-sided *p* < 0.05 was considered statistically significant.

All fNIRS data were processed in MATLAB R2014b (MathWorks, USA) using HomER2, NIRS-SPM, and FC-NIRS. The preprocessing pipeline included conversion of raw intensity signals to optical density and band-pass filtering (0.01–0.10 Hz) to attenuate physiological and low-frequency noise. Optical density signals were converted to concentration changes in HbO and HbR using the modified Beer–Lambert law. Given its higher signal-to-noise ratio relative to HbR, HbO was used as the primary outcome in subsequent analyses. For resting-state fNIRS, time-series HbO signals were extracted at each time point, and Pearson correlation coefficients (r) were computed between channels (and corresponding regions) to quantify whole-brain resting-state functional connectivity. To facilitate parametric statistical testing, correlation coefficients were transformed using Fisher’s *z* transformation. For the picture-naming task, task-related cortical activation was estimated using a general linear model (GLM) applied to the HbO signal of each channel. The model incorporated a canonical hemodynamic response function (HRF), and the resulting *β* coefficients were used as indices of activation strength associated with picture naming. Statistical significance was defined as *p* < 0.05 after Bonferroni correction, with results required to remain significant following correction. Associations between neuroimaging metrics and behavioral/clinical measures were assessed using Pearson correlation when assumptions of normality and homoscedasticity were satisfied; otherwise, Spearman rank correlation was used.

## Results

### Behavioral outcomes

**Figure 3:**
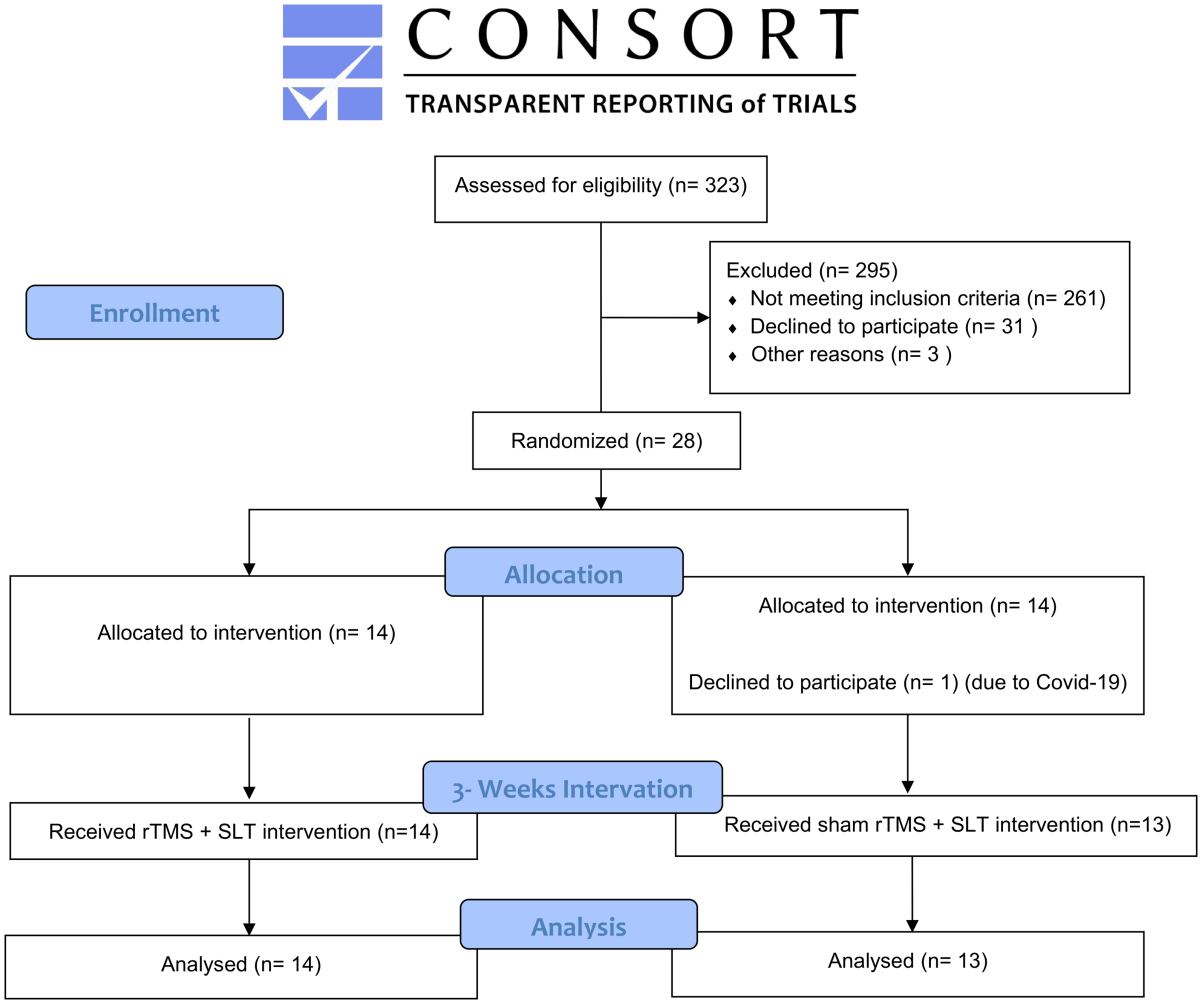
Flow chart for participant selection and assignment.

A total of 323 hospitalized patients were screened for eligibility, of whom 28 were enrolled in the trial. One patient withdrew before the first treatment session because of a severe COVID-19 infection, and the remaining 27 participants completed the full protocol. Baseline demographic and clinical characteristics of all participants are summarized in Table 1; no significant between-group differences were observed in age, sex, stroke type, years of education, cognitive scores, or time since stroke onset (Lesion information and aphasia type are provided in Supplementary Table 1). The trial is reported in accordance with CONSORT guidelines, and the participant flow diagram is shown in Figure 3.

**Table 1:** Baseline demographic and clinical characteristics of the patients.

Characteristics	rTMS (*n* = 14)	Sham (*n* = 13)	*p* Value
Male/female, *n* (%)	10 (71.4%)/4 (28.6%)	9 (69.2%)/4 (30.8%)	0.79
Ischemic/hemorrhagic, *n* (%)	10 (71.4%)/4 (28.6%)	10 (76.9%)/3 (23.1%)	0.77
Age (years)/mean ± SD	56.79 ± 9.23	57.92 ± 8.23	0.73
Time from the onset, days	104.43 ± 34.74	95.77 ± 34.75	0.52
Education, years	9.00 ± 3.03	7.46 ± 3.18	0.06
NACL	76.71 ± 2.79	75.77 ± 2.45	0.36

NLCA, Non-language-based Cognitive Assessment.

### Behavioral outcomes

Table 2 presents the descriptive statistics for all outcome variables at baseline and follow-up for both groups. At baseline, no significant between-group differences were observed for any outcome variable (all *p* > 0.05), confirming successful randomization. In the real rTMS group (the precise anatomical distribution and coordinate locations of the selected stimulation targets in the rTMS group are provided in Supplementary Table 2), 8/14 (57%) participants guessed they had received real rTMS, while 6/14 (43%) guessed sham stimulation; in the sham group, 6/13 (46%) guessed real rTMS, and 7/13 (54%) guessed sham stimulation, the Yates’ continuity-corrected chi-square test indicated successful blinding (*χ*^2^ = 0.017, df = 1, *p* = 0.896).

**Table 2:** Behavioral outcomes at each time point.

Outcome	Time point	rTMS (*N* = 14)	Sham (*N* = 13)
BNT	Pre	10.71 ± 5.78	9.15 ± 2.61
	Post	15.36 ± 8.73	9.85 ± 2.41
	Post–Pre	4.64 ± 3.34	0.69 ± 1.25
Reaction time (ms)	Pre	3,346.50 ± 67.96	3,414.23 ± 173.97
	Post	3,252.21 ± 78.34	3,304.69 ± 67.66
	Post–Pre	−94.29 ± 109.68	−109.54 ± 160.56
Accuracy (%)	Pre	27.93 ± 9.28	31.23 ± 4.80
	Post	36.36 ± 10.84	32.15 ± 5.19
	Post–Pre	8.43 ± 5.81	0.92 ± 1.66
Total AQ	Pre	64.07 ± 16.53	58.17 ± 13.67
	Post	68.74 ± 17.43	61.91 ± 13.52
	Post–Pre	4.67 ± 3.22	3.74 ± 3.93
AQ Spon	Pre	12.79 ± 4.15	10.92 ± 3.23
	Post	13.43 ± 3.92	11.46 ± 3.43
	Post–Pre	0.64 ± 0.93	0.54 ± 0.88
AQ Comp	Pre	8.16 ± 1.23	7.40 ± 1.00
	Post	8.30 ± 1.22	7.46 ± 1.12
	Post–Pre	0.14 ± 0.26	0.06 ± 0.28
AQ Rep	Pre	5.96 ± 2.32	5.65 ± 2.97
	Post	6.28 ± 2.46	6.45 ± 2.55
	Post–Pre	0.32 ± 0.42	0.80 ± 1.46
AQ Naming	Pre	5.14 ± 2.00	5.11 ± 1.73
	Post	6.36 ± 2.34	5.58 ± 1.82
	Post–Pre	1.23 ± 1.11	0.47 ± 0.70

BNT, Boston Naming Test; AQ, aphasia quotient; Spon, Spontaneous speech; Comp, Comprehension; Rep, Repetition.

**Figure 4:**
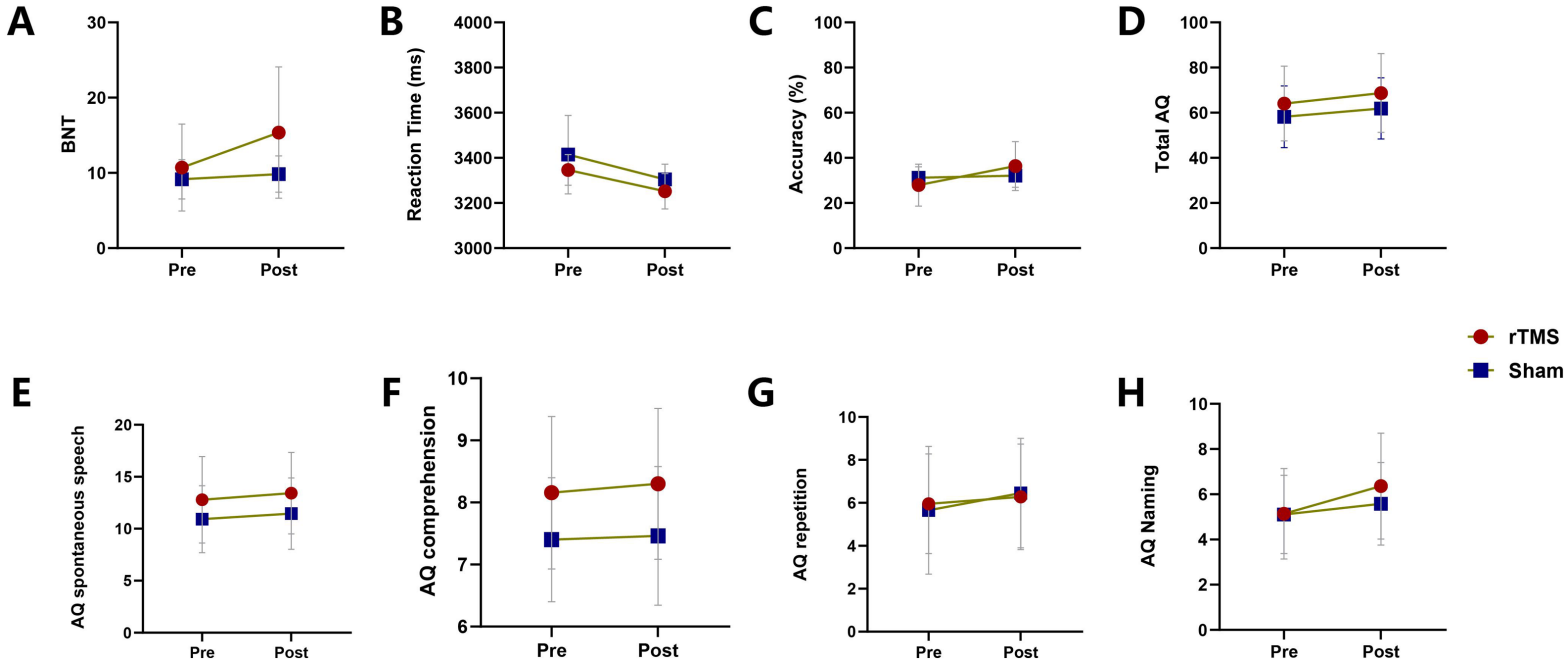
Comparison of longitudinal changes in clinical outcomes between the rTMS group and the sham group; AQ, aphasia quotient.

As shown in Table 3, the primary outcome (Chinese version of the BNT) demonstrated a significant main effect of Time in both the rTMS and sham groups, indicating improvement over the intervention period (*F*[1,25] = 29.25, *p* < 0.001). The main effect of Group was not significant (*F*[1,25] = 2.82, *p* = 0.106), whereas a significant Time × Group interaction was observed (*F*[1,25] = 16.04, *p* < 0.001), suggesting that confrontation naming improved in both groups but with a greater magnitude of recovery in the rTMS group (Figure 4A).

**Table 3:** Summary of results of repeated measures ANOVA.

Outcome	Source of variation	*F*(1,25)	*p*	Partial *η*^2^
BNT	Group	2.82	0.106	0.10
	Time	29.25	<0.001***	0.54
	Group × time	16.04	<0.001***	0.39
Rraction time (ms)	Group	3.75	0.064	0.13
	Time	15.03	<0.001***	0.38
	Group × time	0.08	0.774	0.00
Accuracy (%)	Group	0.02	0.881	0.00
	Time	31.20	<0.001***	0.56
	Group × time	20.10	<0.001***	0.45
Total AQ	Group	1.16	0.292	0.04
	Time	37.21	<0.001***	0.60
	Group × time	0.46	0.505	0.02
AQ Spon	Group	1.82	0.190	0.07
	Time	11.50	0.002**	0.32
	Group × time	0.09	0.767	0.00
AQ Comp	Group	3.31	0.081	0.12
	Time	3.90	0.059	0.14
	Group × time	0.62	0.440	0.02
AQ Rep	Group	0.00	0.948	0.00
	Time	7.64	0.011*	0.23
	Group × time	1.39	0.249	0.05
AQ Naming	Group	0.30	0.590	0.01
	Time	22.20	<0.001***	0.47
	Group × time	4.44	0.045*	0.15

BNT, Boston Naming Test; AQ, aphasia quotient; Spon, Spontaneous speech; Comp, Comprehension; Rep, Repetition; *p < 0.05; **p < 0.01; ***p < 0.001.

For secondary behavioral indices derived from the fNIRS picture-naming task, reaction time showed a significant main effect of Time (*F*[1,25] = 15.03, *p* < 0.001), but neither a significant Group effect (*F*[1,25] = 3.75, *p* = 0.064) nor a Time × Group interaction (*F*[1,25] = 0.08, *p* = 0.774). Naming accuracy also improved significantly over time (*F*[1,25] = 31.20, *p* < 0.001), with no significant main effect of Group (*F*[1,25] = 0.02, *p* = 0.881); however, a significant Time × Group interaction emerged (*F*[1,25] = 20.10, *p* < 0.001), indicating a larger improvement in accuracy in the rTMS group relative to sham (Figure 4B), while between-group differences in reaction time were not evident (Figure 4C). Regarding WAB-R outcomes, significant main effects of Time were observed for the Aphasia Quotient (AQ; *F*[1,25] = 37.21, *p* < 0.001), spontaneous speech (*F*[1,25] = 11.50, *p* = 0.002), repetition (*F*[1,25] = 7.64, *p* = 0.011), and naming (*F*[1,25] = 22.20, *p* < 0.001), whereas improvement in auditory comprehension did not reach statistical significance (*F*[1,25] = 3.90, *p* = 0.059). No significant main effects of Group were detected for any WAB-R subscore (*p* > 0.05). Among the Time × Group interactions, only the naming subscore showed a significant interaction (*F*[1,25] = 4.44, *p* = 0.045), indicating that overall language function improved in both groups (Figures 4D–G), with a greater gain in naming in the rTMS group (Figure 4H).

As a sensitivity analysis (Table 4), separate ANCOVA models with baseline outcome values, AQ, and education years as covariates confirmed that the rTMS group showed a statistically significant improvement in the BNT score compared with the sham group (adjusted mean difference [AMD] = 2.68 [95% CI: 0.66–4.70]; *p* = 0.012; *η*^2^_p_ = 0.26, indicating a large treatment effect). The rTMS group also demonstrated statistically significant improvements in accuracy on the fNIRS picture-naming task (AMD = 8.28 [95% CI: 4.06–12.49]; *p* < 0.001) and in the naming subscore of the WAB-R (AMD = 0.98 [95% CI: 0.10–1.86]; *p* = 0.031). These findings were consistent with the primary RM-ANOVA results, thereby supporting the robustness of the observed treatment effects.

**Table 4:** Summary of results of ANCOVA.

Outcome	rTMS (*N* = 14) Adjusted mean (SE)	Sham (*N* = 13) Adjusted mean (SE)	AMD (95% CI)	Partial *η*^2^	*p*
BNT	13.99 (0.60)	11.31 (0.63)	2.68 (0.66–4.70)	0.26	0.012*
Reaction time (ms)	3,257.87 (21.87)	3,298.60 (22.80)	−40.73 (−110.33 to 28.87)	0.06	0.238
Accuracy (%)	38.32 (1.31)	30.04 (1.37)	8.28 (4.06–12.49)	0.43	0.001**
Total AQ	64.50 (1.39)	66.47 (1.47)	−1.97 (−7.14 to 3.20)	0.03	0.438
AQ Spon	12.36 (0.32)	12.61 (0.34)	−0.24 (−1.40 to 0.92)	0.01	0.669
AQ Comp	7.93 (0.09)	7.86 (0.09)	0.07 (−0.21 to 0.35)	0.01	0.604
AQ Rep	6.21 (0.31)	6.53 (0.33)	−0.32 (−1.37 to 0.73)	0.02	0.537
AQ Naming	6.46 (0.27)	5.48 (0.29)	0.98 (0.10–1.86)	0.20	0.031*

BNT, Boston Naming Test; AQ, aphasia quotient; Spon, Spontaneous speech; Comp, Comprehension; Rep, Repetition; AMD, adjusted mean difference; *p < 0.05; **p < 0.01.

Collectively, behavioral assessments demonstrated significant pre–post improvements in language performance in both groups; however, compared with sham stimulation, active rTMS yielded greater benefits in confrontation naming (BNT), picture-naming accuracy, and WAB-R naming performance. In contrast, no additional advantage of rTMS was observed for picture-naming reaction time.

### Changes in cortical activation and functional connectivity

**Figure 5:**
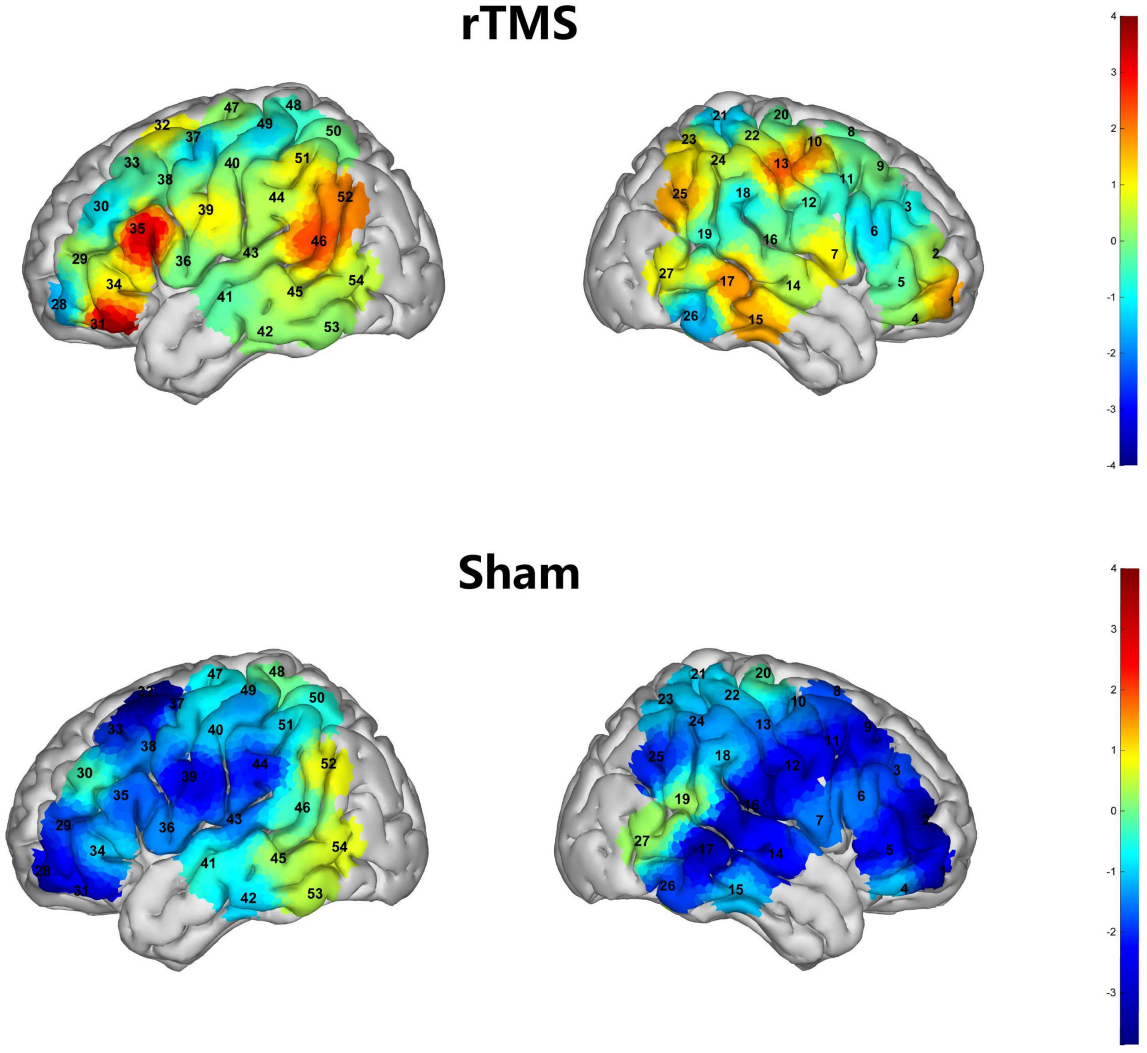
Pre–post changes in task-evoked cortical activation during the picture-naming task measured by fNIRS, shown separately for the rTMS and sham groups; significant channels are reported after FDR correction.

**Figure 6:**
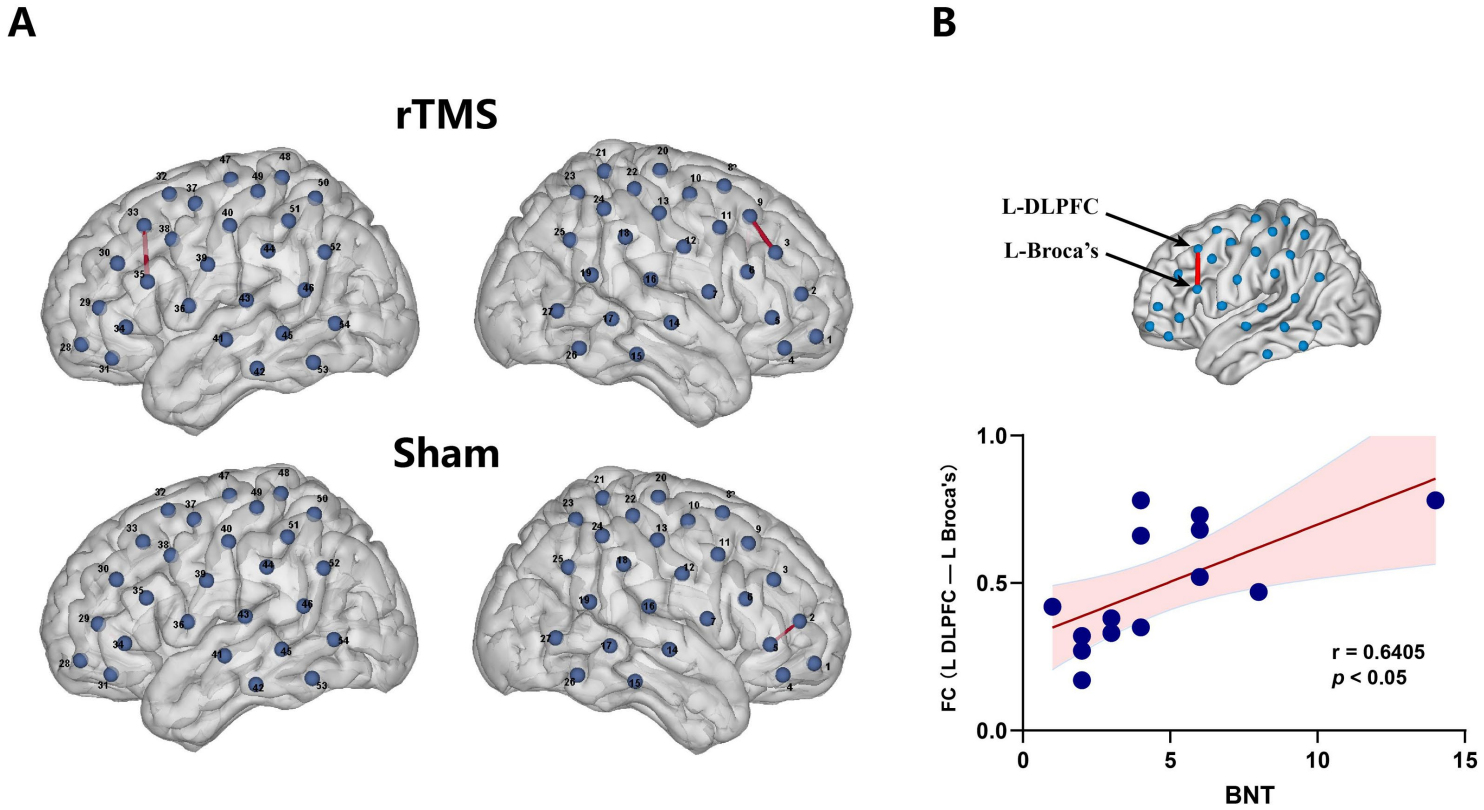
**(A)** Resting-state functional connectivity changes from baseline to post-intervention in the rTMS and sham groups, with significant connections reported after FDR correction. **(B)** In the rTMS group, the pre–post increase in resting-state functional connectivity between the left DLPFC and left Broca’s area was positively associated with improvement in BNT performance.

In the rTMS group, pre–post task-based fNIRS analyses revealed significant increases in activation following treatment in CH31 within the left DLPFC (*T* = 3.62, *p* = 0.03, FDR-corrected) and CH35 within the left L (*T* = 3.39, *p* = 0.03, FDR-corrected). In contrast, no channels showed significant pre–post activation changes in the sham group (Figure 5). Resting-state analyses indicated that, in the rTMS group, functional connectivity was significantly strengthened between CH33 (left DLPFC) and CH35 (left Broca’s area) (*T* = 2.43, *p* = 0.03, FDR-corrected). In addition, connectivity increased between CH9 (right DLPFC) and CH3 (right Broca’s area) (*T* = 3.15, *p* = 0.01, FDR-corrected) (Figure 6A). In the sham group, a significant increase in functional connectivity was observed only between CH2 (right DLPFC) and CH5 (right Broca’s area) (*T* = 2.14, *p* = 0.02, FDR-corrected) (Figure 6A). Collectively, these findings suggest that both groups exhibited enhanced right DLPFC–right Broca’s connectivity after treatment, whereas the rTMS group demonstrated additional left-hemispheric network strengthening (left DLPFC–left Broca’s connectivity) accompanied by increased task-evoked activation during picture naming. To further relate neurophysiological changes to behavioral improvement, correlation analyses were conducted between changes in language outcomes and fNIRS metrics. In the rTMS group, the pre–post change in intra-regional functional connectivity within the left inferior frontal gyrus was positively associated with the corresponding change in BNT performance (*r* = 0.6405, *p* < 0.05; Figure 6B). No other significant associations were found between neuroimaging indices and behavioral language measures.

### Safety

No serious adverse events occurred during the trial. No participants reported dizziness, pain, or seizure-related symptoms.

## Discussion

This study demonstrates the efficacy and safety of fNIRS-guided rTMS targeting individualized picture-naming task activation to treat post-stroke aphasia. Compared with sham stimulation, active rTMS induced a faster and greater recovery of naming ability, yielding larger improvements on naming-centered outcomes, including the Chinese Boston Naming Test (BNT), picture-naming accuracy, and the naming subscore of the Western Aphasia Battery–Revised (WAB-R). Moreover, fNIRS results indicated that this individualized targeting approach significantly increased task-evoked activation in the left dorsolateral prefrontal cortex (DLPFC) and left Broca’s area, and strengthened resting-state functional connectivity between these regions; importantly, the connectivity enhancement was associated with improvements in confrontation naming performance.

Our findings indicate that both groups exhibited significant time-dependent improvements in language performance after the intervention, encompassing nearly all language-scale scores. This pattern is consistent with spontaneous neurological recovery in the subacute post-stroke period as well as the therapeutic effects of intensive speech-language therapy. Notably, the sham group also demonstrated measurable language recovery throughout the treatment course. This may be explained by the fact that many enrolled patients were in the subacute stage, during which language recovery is typically most rapid in the first few months after stroke and then gradually attenuates, reaching a relatively stable plateau after approximately 6 months as patients transition into the chronic stage (Kertesz and McCabe, 1977; Sul et al., 2016). In the present cohort, the mean time since onset was approximately 3 months, corresponding to the early-to-mid subacute phase, suggesting that participants were recruited during a critical window characterized by heightened neuroplastic potential (Schevenels et al., 2022; Yan et al., 2025). We further observed an intriguing neurophysiological pattern: after intervention, both groups showed a significant increase in functional connectivity between the right-hemispheric dorsolateral prefrontal cortex (DLPFC) and the right Broca’s area. This change may reflect spontaneous recovery, the effects of intensive speech-language therapy, or their combined influence, although these contributions are difficult to disentangle. In a large-sample study, Saur and colleagues reported that recovery of language function in subacute aphasia is accompanied by more pronounced activation within frontal regions of both hemispheres, potentially reflecting resolution of acute network disruption and re-engagement of preserved language-related networks (Saur et al., 2006). In addition, resting-state investigations have shown that aphasia severity is closely associated with widespread disruptions in bilaterally distributed brain networks (Zhu et al., 2014; Klingbeil et al., 2019). In our previous resting-state fMRI study, we likewise demonstrated that increased right frontal activity in patients with early-stage aphasia was associated with spontaneous language recovery (Li et al., 2023).

Active rTMS conferred greater benefits on naming-centered outcomes (BNT, picture-naming accuracy, and the WAB-R naming subscore), supporting the notion that fNIRS-guided, individualized targeting based on picture-naming task activation can meaningfully enhance naming performance in post-stroke aphasia. Anomia is among the most prevalent and persistent language deficits in aphasia (Goodglass and Wingfield, 1997), and naming performance has been proposed as a clinically informative indicator of language recovery potential (Meier et al., 2020). From a neuroplasticity perspective, earlier intervention is generally considered more likely to yield larger effects in aphasic (Laska et al., 2001), particularly during the subacute period when the brain exhibits heightened responsiveness to neuromodulatory inputs. Preclinical evidence is consistent with this time-sensitive framework: repetitive transcranial magnetic stimulation administered in the acute phase has been shown in rodent ischemic stroke models to improve neurological function and reduce lesion-related pathology (Luo et al., 2022; Zhang et al., 2025). In clinical subacute aphasia, mechanistic observations further suggest a biological basis for recovery facilitation; for example, Bai and colleagues reported that rTMS combined with conventional speech training was accompanied by increases in serum brain-derived neurotrophic factor (BDNF), implicating neurotrophic signaling pathways in treatment-related gains (Bai et al., 2020; Bai et al., 2022). Moreover, meta-analytic evidence indicates that rTMS tends to yield more robust effects in subacute post-stroke aphasia than in chronic stages, where effect sizes are often smaller to moderate and more variable (Chai et al., 2024). Consistent with this stage-dependent pattern, the multicenter, multilingual NORTHSTAR-CA trial reported that adding rTMS to speech-language therapy produced supplemental gains in naming in the subacute phase, whereas comparable benefits were not observed to the same extent in chronic aphasia, highlighting the greater recovery potential during the subacute window (Zumbansen et al., 2022).

Post-stroke interhemispheric imbalance, mediated in part by transcallosal inhibition, represents an influential framework for understanding language impairment and recovery in aphasia (Chrysikou and Hamilton, 2011). Within this model, the nondominant hemisphere may exert a maladaptive influence: release from transcallosal inhibition can lead to increased contralesional activity, which may in turn suppress language-related processing in the lesioned dominant hemisphere (Hamilton et al., 2011). Saur’s three-stage model described a dynamic evolution in activation within the left-dominant language network: diminished and weak left-hemisphere activation in the acute stage, increased bilateral activation in the subacute stage, and subsequent re-lateralization toward the left hemisphere in the chronic stage, accompanying substantial recovery in many patients (Saur et al., 2006). In the present study, we intervened during the subacute stage by stimulating the individually most strongly activated cortical site during picture naming, a strategy that may facilitate the transition from a subacute, bilaterally engaged configuration toward a more left-dominant organization. Consistent with this interpretation, our post-intervention fNIRS results showed robust task-evoked activation and strengthened functional connectivity within the left hemisphere during picture naming. This pattern is convergent with prior evidence indicating that early aphasia rehabilitation is associated with reactivation of left-hemispheric language-related regions (Mattioli et al., 2014). Moreover, multiple studies have reported that excitatory, high-frequency rTMS applied to the left hemisphere can promote recruitment of residual ipsilesional language resources in post-stroke aphasia (Szaflarski et al., 2011; Dammekens et al., 2014; Griffis et al., 2016; Szaflarski et al., 2018), potentially supporting synaptic reorganization and improvements in cortical function (Allendorfer et al., 2012). This interpretation is also consistent with Hamilton et al., who proposed that noninvasive brain stimulation may play a pivotal role in improving naming during aphasia rehabilitation, potentially by engaging multiple residual language-related networks supporting naming within the left hemisphere and thereby contributing to broader language gains (Rubi-Fessen et al., 2015). In an early prospective study, stimulation guided by functionally mapped sites was likewise found to yield immediate improvements in language performance after intervention, plausibly by facilitating reintegration of residual left-hemispheric cortex into critical language networks (Hartwigsen and Saur, 2019). Relatedly, stimulation-induced reversal of atypical language lateralization has been proposed to support the dynamic processes underlying spontaneous recovery in aphasia (Allendorfer et al., 2021), which is consistent with our observation of a pronounced post-intervention increase in left frontal activation during picture naming in the rTMS group, indicating strengthened ipsilesional dominance. Resting-state connectivity findings provide additional context. Sandberg (2017) reported widespread reductions in both intra- and interhemispheric functional connectivity in aphasia relative to healthy controls, suggesting a general tendency toward decreased intrinsic connectivity; importantly, connectivity strength has also been linked to aphasia severity. These observations are compatible with the notion that stroke disrupts resting-state networks and that treatment-related strengthening of functional connectivity may be associated with clinical benefit (Gili et al., 2017). Supporting this perspective, Dammekens et al. (2014) described a patient with nonfluent aphasia who received high-frequency rTMS over the left inferior frontal gyrus; EEG analyses demonstrated increased theta- and beta3-band connectivity between bilateral inferior frontal regions. This is broadly concordant with our neuroimaging findings, in which high-frequency stimulation delivered within the left frontal system was accompanied by increased bilateral frontal connectivity and was associated with improved naming. Finally, the neurocognitive architecture of naming offers a plausible basis for the prominent involvement of the DLPFC observed in our data. Naming is a complex operation encompassing multiple partially dissociable cognitive processes and representational levels (Levelt, 2001; Indefrey, 2011). The DLPFC is commonly implicated in higher-order cognitive control and has been increasingly recognized as contributing to language production (Brunoni and Vanderhasselt, 2014; Hertrich et al., 2021), which may help explain why, after rTMS, the left DLPFC exhibited robust task-evoked activation and stronger functional coupling with the left Broca’s region in our cohort.

From a clinical perspective, the present findings suggest that rTMS guided by individualized fNIRS activation mapping during a picture-naming task is a safe and effective intervention for improving naming deficits in patients with subacute post-stroke aphasia. However, several limitations should be acknowledged. First, the relatively small sample size and single-center design limited subgroup analyses and may restrict the generalizability of the findings, highlighting the need for larger, multicenter randomized controlled trials. Second, the lack of long-term follow-up prevents conclusions regarding the durability of treatment effects; due to COVID-19-related restrictions in Shanghai during the study period, longitudinal assessments were not feasible. Although most patients reported subjective improvement at discharge, these observations were not supported by systematic data collection or statistical validation, leaving the long-term maintenance of therapeutic effects uncertain. Third, this study relied solely on fNIRS for neuroimaging guidance, given its limited spatial resolution and inability to capture lesion-specific information, the precision of neurophysiological interpretation may be constrained. Our future studies will incorporate multimodal neuroimaging approaches and standardized long-term follow-up to further optimize the protocol and enhance the reliability and validity of the findings.

## Conclusion

This study presents an effective and safe fNIRS-guided rTMS protocol whereby individualized stimulation targets are determined based on task-evoked brain activation during picture naming. This intervention demonstrates a potential short-term beneficial effect on picture-naming performance in patients with subacute post-stroke aphasia, yet its long-term efficacy remains to be further verified. Moreover, this neuroimaging-guided stimulation significantly enhances task-related activation in the left DLPFC and left Broca’s area, and strengthens the functional connectivity between the left DLPFC and Broca’s region.

## Data Availability

The raw data supporting the conclusions of this article will be made available by the authors without undue reservation.

## References

[ref1] Allendorfer J. B. Nenert R. Vannest J. Szaflarski J. P. (2021). A pilot randomized controlled trial of intermittent theta burst stimulation as stand-alone treatment for post-stroke aphasia: effects on language and verbal functional magnetic resonance imaging (fMRI). Med. Sci. Monit. 27:e934818. doi: 10.12659/msm.934818, 34862359 PMC8653428

[ref2] Allendorfer J. B. Storrs J. M. Szaflarski J. P. (2012). Changes in white matter integrity follow excitatory rTMS treatment of post-stroke aphasia. Restor. Neurol. Neurosci. 30, 103–113. doi: 10.3233/rnn-2011-0627, 22233802 PMC3316910

[ref3] Bai G. Jiang L. Huan S. Meng P. Wang Y. Pan X. . (2022). Study on low-frequency repetitive transcranial magnetic stimulation improves speech function and mechanism in patients with non-fluent aphasia after stroke. Front. Aging Neurosci. 14:883542. doi: 10.3389/fnagi.2022.883542, 35711903 PMC9197107

[ref4] Bai G. Jiang L. Ma W. Meng P. Li J. Wang Y. . (2020). Effect of low-frequency rTMS and intensive speech therapy treatment on patients with nonfluent aphasia after stroke. Neurologist 26, 6–9. doi: 10.1097/nrl.0000000000000303, 33394904

[ref5] Bai Z. Zhang J. Fong K. N. K. (2022). Effects of transcranial magnetic stimulation in modulating cortical excitability in patients with stroke: a systematic review and meta-analysis. J. Neuroeng. Rehabil. 19:24. doi: 10.1186/s12984-022-00999-4, 35193624 PMC8862292

[ref6] Brunoni A. R. Vanderhasselt M. A. (2014). Working memory improvement with non-invasive brain stimulation of the dorsolateral prefrontal cortex: a systematic review and meta-analysis. Brain Cogn. 86, 1–9. doi: 10.1016/j.bandc.2014.01.008, 24514153

[ref7] Burton B. Isaacs M. Brogan E. Shrubsole K. Kilkenny M. F. Power E. . (2023). An updated systematic review of stroke clinical practice guidelines to inform aphasia management. Int. J. Stroke 18, 1029–1039. doi: 10.1177/17474930231161454, 36803248 PMC10614176

[ref8] Butler L. K. Kiran S. Tager-Flusberg H. (2020). Functional near-infrared spectroscopy in the study of speech and language impairment across the life span: a systematic review. Am. J. Speech Lang. Pathol. 29, 1674–1701. doi: 10.1044/2020_ajslp-19-00050, 32640168 PMC7893520

[ref9] Chai L. Huang Y. Guo X. Xiong A. Lin B. Huang J. (2024). Does SLT combined with NIBS enhance naming recovery in post-stroke aphasia? A meta-analysis and systematic review. NeuroRehabilitation 54, 543–561. doi: 10.3233/nre-240065, 38875053 PMC11307008

[ref10] Chang W. K. Park J. Lee J. Y. Cho S. Lee J. Kim W. S. . (2022). Functional network changes after high-frequency rTMS over the most activated speech-related area combined with speech therapy in chronic stroke with non-fluent aphasia. Front. Neurol. 13:690048. doi: 10.3389/fneur.2022.690048, 35222235 PMC8866644

[ref11] Chen T. B. Lin C. Y. Lin K. N. Yeh Y. C. Chen W. T. Wang K. S. . (2014). Culture qualitatively but not quantitatively influences performance in the Boston naming test in a Chinese-speaking population. Dement. Geriatr. Cogn. Dis. Extra 4, 86–94. doi: 10.1159/000360695, 24847347 PMC4024970

[ref12] Chen Q. Shen W. Sun H. Zhang H. Liu C. Chen Z. . (2022). The effect of coupled inhibitory-facilitatory repetitive transcranial magnetic stimulation on shaping early reorganization of the motor network after stroke. Brain Res. 1790:147959. doi: 10.1016/j.brainres.2022.147959, 35654120

[ref13] Chen W. L. Wagner J. Heugel N. Sugar J. Lee Y. W. Conant L. . (2020). Functional near-infrared spectroscopy and its clinical application in the field of neuroscience: advances and future directions. Front. Neurosci. 14:724. doi: 10.3389/fnins.2020.00724, 32742257 PMC7364176

[ref14] Chrysikou E. G. Hamilton R. H. (2011). Noninvasive brain stimulation in the treatment of aphasia: exploring interhemispheric relationships and their implications for neurorehabilitation. Restor. Neurol. Neurosci. 29, 375–394. doi: 10.3233/rnn-2011-0610, 22124035

[ref15] Chung S. W. Hill A. T. Rogasch N. C. Hoy K. E. Fitzgerald P. B. (2016). Use of theta-burst stimulation in changing excitability of motor cortex: a systematic review and meta-analysis. Neurosci. Biobehav. Rev. 63, 43–64. doi: 10.1016/j.neubiorev.2016.01.008, 26850210

[ref16] Dammekens E. Vanneste S. Ost J. De Ridder D. (2014). Neural correlates of high frequency repetitive transcranial magnetic stimulation improvement in post-stroke non-fluent aphasia: a case study. Neurocase 20, 1–9. doi: 10.1080/13554794.2012.713493, 22963195

[ref17] Dang H. Chen J. Li J. Wu Y. Zeng Z. (2025). Effects of speech rehabilitation training combined with repetitive transcranial magnetic stimulation on patients with post-stroke aphasia. Clin. Neurol. Neurosurg. 252:108833. doi: 10.1016/j.clineuro.2025.108833, 40120284

[ref18] Doucet T. Muller F. Verdun-Esquer C. Debelleix X. Brochard P. (2012). Returning to work after a stroke: a retrospective study at the physical and rehabilitation medicine center La Tour de Gassies. Ann. Phys. Rehabil. Med. 55, 112–127. doi: 10.1016/j.rehab.2012.01.007, 22386687

[ref19] Du J. Yang F. Hu J. Hu J. Xu Q. Cong N. . (2019). Effects of high- and low-frequency repetitive transcranial magnetic stimulation on motor recovery in early stroke patients: evidence from a randomized controlled trial with clinical, neurophysiological and functional imaging assessments. Neuroimage Clin. 21:101620. doi: 10.1016/j.nicl.2018.101620, 30527907 PMC6411653

[ref20] Ellis C. Simpson A. N. Bonilha H. Mauldin P. D. Simpson K. N. (2012). The one-year attributable cost of poststroke aphasia. Stroke 43, 1429–1431. doi: 10.1161/strokeaha.111.647339, 22343643 PMC4507407

[ref21] Fang D. Ji X. Li H. Xu S. Yang Y. Zhan J. . (2025). Fixel-based white matter correlates of sentence comprehension in post-stroke aphasia. Brain Sci. 15:1039. doi: 10.3390/brainsci15101039, 41154135 PMC12564056

[ref22] Feng J. Hu R. Lyu M. Ma X. Li T. Meng Y. . (2025). Right C7 neurotomy at the intervertebral foramen plus intensive speech and language therapy versus intensive speech and language therapy alone for chronic post-stroke aphasia: multicentre, randomised controlled trial. BMJ 389:e083605. doi: 10.1136/bmj-2024-083605, 40562426 PMC12189313

[ref23] Gan L. Huang L. Zhang Y. Yang X. Li L. Meng L. . (2024). Effects of low-frequency rTMS combined with speech and language therapy on Broca's aphasia in subacute stroke patients. Front. Neurol. 15:1473254. doi: 10.3389/fneur.2024.1473254, 39539660 PMC11557360

[ref24] Gerstenecker A. Lazar R. M. (2019). Language recovery following stroke. Clin. Neuropsychol. 33, 928–947. doi: 10.1080/13854046.2018.1562093, 30698070 PMC8985654

[ref25] Gholami M. Pourbaghi N. Taghvatalab S. (2022). Evaluation of rTMS in patients with poststroke aphasia: a systematic review and focused meta-analysis. Neurol. Sci. 43, 4685–4694. doi: 10.1007/s10072-022-06092-x, 35499630

[ref26] Gili T. Fiori V. De Pasquale G. Sabatini U. Caltagirone C. Marangolo P. (2017). Right sensory-motor functional networks subserve action observation therapy in aphasia. Brain Imaging Behav. 11, 1397–1411. doi: 10.1007/s11682-016-9635-1, 27734301

[ref27] Goodglass H. Wingfield A. (1997). Anomia: Neuroanatomical and Cognitive Correlates. San Diego, CA: Academic Press.

[ref28] Griffis J. C. Nenert R. Allendorfer J. B. Szaflarski J. P. (2016). Interhemispheric plasticity following intermittent theta burst stimulation in chronic poststroke aphasia. Neural Plast. 2016, 1–16. doi: 10.1155/2016/4796906, 26881111 PMC4736997

[ref29] Hamilton R. H. Chrysikou E. G. Coslett B. (2011). Mechanisms of aphasia recovery after stroke and the role of noninvasive brain stimulation. Brain Lang. 118, 40–50. doi: 10.1016/j.bandl.2011.02.005, 21459427 PMC3109088

[ref30] Hara T. Abo M. Kakita K. Mori Y. Yoshida M. Sasaki N. (2017). The effect of selective transcranial magnetic stimulation with functional near-infrared spectroscopy and intensive speech therapy on individuals with post-stroke aphasia. Eur. Neurol. 77, 186–194. doi: 10.1159/000457901, 28161706

[ref31] Hartwigsen G. Saur D. (2019). Neuroimaging of stroke recovery from aphasia - insights into plasticity of the human language network. NeuroImage 190, 14–31. doi: 10.1016/j.neuroimage.2017.11.056, 29175498

[ref32] Hermann D. M. Chopp M. (2012). Promoting brain remodelling and plasticity for stroke recovery: therapeutic promise and potential pitfalls of clinical translation. Lancet Neurol. 11, 369–380. doi: 10.1016/s1474-4422(12)70039-x, 22441198 PMC3964179

[ref33] Hertrich I. Dietrich S. Blum C. Ackermann H. (2021). The role of the dorsolateral prefrontal cortex for speech and language processing. Front. Hum. Neurosci. 15:645209. doi: 10.3389/fnhum.2021.645209, 34079444 PMC8165195

[ref34] Indefrey P. (2011). The spatial and temporal signatures of word production components: a critical update. Front. Psychol. 2:255. doi: 10.3389/fpsyg.2011.00255, 22016740 PMC3191502

[ref35] Irani F. Platek S. M. Bunce S. Ruocco A. C. Chute D. (2007). Functional near infrared spectroscopy (fNIRS): an emerging neuroimaging technology with important applications for the study of brain disorders. Clin. Neuropsychol. 21, 9–37. doi: 10.1080/13854040600910018, 17366276

[ref36] Juan D. Yao W. Li J. Yang F. Hu J. Xu Q. . (2022). Motor network reorganization after repetitive transcranial magnetic stimulation in early stroke patients: a resting state fMRI study. Neurorehabil. Neural Repair 36, 61–68. doi: 10.1177/15459683211054184, 34711080

[ref37] Kakuda W. Abo M. Kaito N. Watanabe M. Senoo A. (2010). Functional MRI-based therapeutic rTMS strategy for aphasic stroke patients: a case series pilot study. Int. J. Neurosci. 120, 60–66. doi: 10.3109/00207450903445628, 20128673

[ref38] Kertesz A. McCabe P. (1977). Recovery patterns and prognosis in aphasia. Brain 100, 1–18. doi: 10.1093/brain/100.1.1, 861709

[ref39] Kielar A. Patterson D. Chou Y. H. (2022). Efficacy of repetitive transcranial magnetic stimulation in treating stroke aphasia: systematic review and meta-analysis. Clin. Neurophysiol. 140, 196–227. doi: 10.1016/j.clinph.2022.04.017, 35606322

[ref40] Klingbeil J. Wawrzyniak M. Stockert A. Saur D. (2019). Resting-state functional connectivity: an emerging method for the study of language networks in post-stroke aphasia. Brain Cogn. 131, 22–33. doi: 10.1016/j.bandc.2017.08.005, 28865994

[ref41] Knopman D. S. Selnes O. A. Niccum N. Rubens A. B. (1984). Recovery of naming in aphasia: relationship to fluency, comprehension and CT findings. Neurology 34, 1461–1470. doi: 10.1212/wnl.34.11.1461, 6493494

[ref42] Laska A. C. Hellblom A. Murray V. Kahan T. Von Arbin M. (2001). Aphasia in acute stroke and relation to outcome. J. Intern. Med. 249, 413–422. doi: 10.1046/j.1365-2796.2001.00812.x, 11350565

[ref43] Lazar R. M. Boehme A. K. (2017). Aphasia as a predictor of stroke outcome. Curr. Neurol. Neurosci. Rep. 17:83. doi: 10.1007/s11910-017-0797-z, 28929424 PMC13077792

[ref44] Levelt W. J. (2001). Spoken word production: a theory of lexical access. Proc. Natl. Acad. Sci. USA 98, 13464–13471. doi: 10.1073/pnas.231459498, 11698690 PMC60894

[ref45] Li H. Fan S. Wu Y. Fang D. Hu R. Lu R. (2024). Intermittent theta-burst stimulation in aphasia caused by right side cerebral lesions after stroke: a case report with 2-year follow-up. Heliyon 10:e35206. doi: 10.1016/j.heliyon.2024.e35206, 39166089 PMC11333899

[ref46] Li X. Y. Hu R. Lou T. X. Liu Y. Ding L. (2024). Global research trends in transcranial magnetic stimulation for stroke (1994-2023): promising, yet requiring further practice. Front. Neurol. 15:1424545. doi: 10.3389/fneur.2024.1424545, 39268062 PMC11390666

[ref47] Li J. Li H. Peng C. Xu W. Chen Q. Liu G. (2024). Paradoxical cognitive and language function recovery by zolpidem in a patient with traumatic brain injury: a case report. Medicine (Baltimore) 103:e38964. doi: 10.1097/md.0000000000038964, 38996115 PMC11245188

[ref48] Li H. Liu J. Tian S. Fan S. Wang T. Qian H. . (2022). Language reorganization patterns in global aphasia-evidence from fNIRS. Front. Neurol. 13:1025384. doi: 10.3389/fneur.2022.1025384, 36686505 PMC9853054

[ref49] Li Y. Qiao Y. Wang F. Wei C. Wang R. Jin H. . (2022). Culture effects on the Chinese version Boston naming test performance and the normative data in the native Chinese-speaking elders in mainland China. Front. Neurol. 13:866261. doi: 10.3389/fneur.2022.866261, 35645954 PMC9139106

[ref50] Li H. Zhang H. Xu S. Wang M. Zhang J. Liu J. . (2023). Altered spontaneous brain activity in Poststroke aphasia: a resting-state fMRI study. Brain Sci. 13:300. doi: 10.3390/brainsci13020300, 36831843 PMC9954170

[ref51] Luo L. Liu M. Fan Y. Zhang J. Liu L. Li Y. . (2022). Intermittent theta-burst stimulation improves motor function by inhibiting neuronal pyroptosis and regulating microglial polarization via TLR4/NFκB/NLRP3 signaling pathway in cerebral ischemic mice. J. Neuroinflammation 19:141. doi: 10.1186/s12974-022-02501-2, 35690810 PMC9188077

[ref52] Ma J. Zhang Z. Su Y. Kang L. Geng D. Wang Y. . (2013). Magnetic stimulation modulates structural synaptic plasticity and regulates BDNF-TrkB signal pathway in cultured hippocampal neurons. Neurochem. Int. 62, 84–91. doi: 10.1016/j.neuint.2012.11.010, 23201339

[ref53] Mattioli F. Ambrosi C. Mascaro L. Scarpazza C. Pasquali P. Frugoni M. . (2014). Early aphasia rehabilitation is associated with functional reactivation of the left inferior frontal gyrus: a pilot study. Stroke 45, 545–552. doi: 10.1161/strokeaha.113.003192, 24309584

[ref54] McDonnell M. N. Stinear C. M. (2017). TMS measures of motor cortex function after stroke: a meta-analysis. Brain Stimul. 10, 721–734. doi: 10.1016/j.brs.2017.03.008, 28385535

[ref55] Meier E. L. Bunker L. D. Kim H. Hillis A. E. (2023). Resting-state connectivity in acute and subacute poststroke aphasia: a functional near-infrared spectroscopy pilot study. Brain Connect. 13, 441–452. doi: 10.1089/brain.2022.0065, 37097208 PMC10618818

[ref56] Meier E. L. Sheppard S. M. Goldberg E. B. Head C. R. Ubellacker D. M. Walker A. . (2020). Naming errors and dysfunctional tissue metrics predict language recovery after acute left hemisphere stroke. Neuropsychologia 148:107651. doi: 10.1016/j.neuropsychologia.2020.107651, 33045231 PMC7546715

[ref57] Ntasiopoulou C. Nasios G. Messinis L. Nousia A. Siokas V. Dardiotis E. (2023). Repetitive transcranial magnetic stimulation in post-stroke aphasia: comparative evaluation of inhibitory and excitatory therapeutic protocols: narrative review. Adv. Exp. Med. Biol. 1425, 619–628. doi: 10.1007/978-3-031-31986-0_60, 37581835

[ref58] Pell G. S. Roth Y. Zangen A. (2011). Modulation of cortical excitability induced by repetitive transcranial magnetic stimulation: influence of timing and geometrical parameters and underlying mechanisms. Prog. Neurobiol. 93, 59–98. doi: 10.1016/j.pneurobio.2010.10.003, 21056619

[ref59] Postman-Caucheteux W. A. Birn R. M. Pursley R. H. Butman J. A. Solomon J. M. Picchioni D. . (2010). Single-trial fMRI shows contralesional activity linked to overt naming errors in chronic aphasic patients. J. Cogn. Neurosci. 22, 1299–1318. doi: 10.1162/jocn.2009.21261, 19413476 PMC4778722

[ref60] Ren J. Ren W. Zhou Y. Dahmani L. Duan X. Fu X. . (2023). Personalized functional imaging-guided rTMS on the superior frontal gyrus for post-stroke aphasia: a randomized sham-controlled trial. Brain Stimul. 16, 1313–1321. doi: 10.1016/j.brs.2023.08.023, 37652135

[ref61] Rubi-Fessen I. Hartmann A. Huber W. Fimm B. Rommel T. Thiel A. . (2015). Add-on effects of repetitive transcranial magnetic stimulation on subacute aphasia therapy: enhanced improvement of functional communication and basic linguistic skills. A randomized controlled study. Arch. Phys. Med. Rehabil. 96, 1935–44.e2. doi: 10.1016/j.apmr.2015.06.017, 26189201

[ref62] Sandberg C. W. (2017). Hypoconnectivity of resting-state networks in persons with aphasia compared with healthy age-matched adults. Front. Hum. Neurosci. 11:91. doi: 10.3389/fnhum.2017.00091, 28293185 PMC5329062

[ref63] Saur D. Lange R. Baumgaertner A. Schraknepper V. Willmes K. Rijntjes M. . (2006). Dynamics of language reorganization after stroke. Brain 129, 1371–1384. doi: 10.1093/brain/awl090, 16638796

[ref64] Schevenels K. Gerrits R. Lemmens R. De Smedt B. Zink I. Vandermosten M. (2022). Early white matter connectivity and plasticity in post stroke aphasia recovery. Neuroimage Clin. 36:103271. doi: 10.1016/j.nicl.2022.103271, 36510409 PMC9723316

[ref65] Sul B. Kim J. S. Hong B. Y. Lee K. B. Hwang W. S. Kim Y. K. . (2016). The prognosis and recovery of aphasia related to stroke lesion. Ann. Rehabil. Med. 40, 786–793. doi: 10.5535/arm.2016.40.5.786, 27847708 PMC5108705

[ref66] Szaflarski J. P. Griffis J. Vannest J. Allendorfer J. B. Nenert R. Amara A. W. . (2018). A feasibility study of combined intermittent theta burst stimulation and modified constraint-induced aphasia therapy in chronic post-stroke aphasia. Restor. Neurol. Neurosci. 36, 503–518. doi: 10.3233/rnn-180812, 29889086

[ref67] Szaflarski J. P. Vannest J. Wu S. W. DiFrancesco M. W. Banks C. Gilbert D. L. (2011). Excitatory repetitive transcranial magnetic stimulation induces improvements in chronic post-stroke aphasia. Med. Sci. Monit. 17, Cr132–Cr139. doi: 10.12659/msm.881446, 21358599 PMC3057942

[ref68] Thiel A. Hartmann A. Rubi-Fessen I. Anglade C. Kracht L. Weiduschat N. . (2013). Effects of noninvasive brain stimulation on language networks and recovery in early poststroke aphasia. Stroke 44, 2240–2246. doi: 10.1161/strokeaha.111.000574, 23813984

[ref69] Waldowski K. Seniów J. Leśniak M. Iwański S. Członkowska A. (2012). Effect of low-frequency repetitive transcranial magnetic stimulation on naming abilities in early-stroke aphasic patients: a prospective, randomized, double-blind sham-controlled study. ScientificWorldJournal 2012:518568. doi: 10.1100/2012/518568, 23213288 PMC3508571

[ref70] Wang Y. Fang K. He S. Fan Y. Yu J. Zhang X. (2019). Effects of repetitive magnetic stimulation on the growth of primarily cultured hippocampus neurons in vitro and their expression of iron-containing enzymes. Neuropsychiatr. Dis. Treat. 15, 927–934. doi: 10.2147/ndt.S199328, 31114204 PMC6489628

[ref71] Wang T. Huang X. Zhao L. Wang Y. Zhang S. Fu X. . (2023). A bibliometric analysis of global publication trends on rTMS and aphasia. Medicine (Baltimore) 102:e33826. doi: 10.1097/md.0000000000033826, 37335693 PMC10194649

[ref72] Winhuisen L. Thiel A. Schumacher B. Kessler J. Rudolf J. Haupt W. F. . (2005). Role of the contralateral inferior frontal gyrus in recovery of language function in poststroke aphasia: a combined repetitive transcranial magnetic stimulation and positron emission tomography study. Stroke 36, 1759–1763. doi: 10.1161/01.STR.0000174487.81126.ef, 16020770

[ref73] Xu A. H. Sun Y. X. (2020). Research hotspots and effectiveness of repetitive transcranial magnetic stimulation in stroke rehabilitation. Neural Regen. Res. 15, 2089–2097. doi: 10.4103/1673-5374.282269, 32394967 PMC7716019

[ref74] Yan W. Lin Y. Chen Y. F. Wang Y. Wang J. Zhang M. (2025). Enhancing neuroplasticity for post-stroke motor recovery: mechanisms, models, and neurotechnology. IEEE Trans. Neural Syst. Rehabil. Eng. 33, 1156–1168. doi: 10.1109/tnsre.2025.3551753, 40100694

[ref75] Yang G. Fan C. Li H. Tong Y. Lin S. Feng Y. . (2025). Resting-state brain network characteristics related to mild cognitive impairment: a preliminary fNIRS proof-of-concept study. J. Integr. Neurosci. 24:26406. doi: 10.31083/jin26406, 40018781

[ref76] Zhang J. Ding M. Luo L. Huang D. Li S. Chen S. . (2025). Intermittent theta-burst stimulation promotes neurovascular unit remodeling after ischemic stroke in a mouse model. Neural Regen. Res. doi: 10.4103/nrr.Nrr-d-24-01189 PMC13452689 40145963

[ref77] Zhang Q. Yang Y. (2003). The determiners of picture-naming latency. Acta Psychol. Sin. 35:447. Available online at: https://journal.psych.ac.cn/xlxb/CN/Y2003/V35/I04/447

[ref78] Zheng K. Xu X. Ji Y. Fang H. Gao F. Huang G. . (2022). Continuous theta burst stimulation-induced suppression of the right fronto-thalamic-cerebellar circuit accompanies improvement in language performance in poststroke aphasia: a resting-state fMRI study. Front. Aging Neurosci. 14:1079023. doi: 10.3389/fnagi.2022.1079023, 36711202 PMC9877515

[ref79] Zhu D. Chang J. Freeman S. Tan Z. Xiao J. Gao Y. . (2014). Changes of functional connectivity in the left frontoparietal network following aphasic stroke. Front. Behav. Neurosci. 8:167. doi: 10.3389/fnbeh.2014.00167, 24860452 PMC4026698

[ref80] Zumbansen A. Kneifel H. Lazzouni L. Ophey A. Black S. E. Chen J. L. . (2022). Differential effects of speech and language therapy and rTMS in chronic versus subacute post-stroke aphasia: results of the NORTHSTAR-CA trial. Neurorehabil. Neural Repair 36, 306–316. doi: 10.1177/15459683211065448, 35337223 PMC9003806

