# Supplementary material for: fNIRS-Guided neuronavigated rTMS augments naming recovery in subacute post-stroke aphasia: a double-blind randomized controlled trial
Source: Front Hum Neurosci. 2026 Apr 22;20:1810169. doi: 10.3389/fnhum.2026.1810169 (PMC13144099; doi:10.3389/fnhum.2026.1810169)
Supplement: Supplementary file 1 [file Table_1.DOCX]

# Supplementary Table 1. Lesion and Aphasia type of each patient

| **Group** | **Patient** | **Lesion** | **Type of Aphasia** |
| --- | --- | --- | --- |
| **rTMS** | **PA1** | **Left frontal and temporal lobes** | **Broca's aphasia** |
| **rTMS** | **PA2** | **Left temporal lobe** | **Anomic aphasia** |
| **rTMS** | **PA3** | **Left frontal, temporal and parietal lobes** | **Anomic aphasia** |
| **rTMS** | **PA4** | **Left temporooccipital lobe and basal ganglia** | **Broca's aphasia** |
| **rTMS** | **PA5** | **Left frontal lobe** | **Broca's aphasia** |
| **rTMS** | **PA6** | **Left frontal and temporal lobes** | **Broca's aphasia** |
| **rTMS** | **PA7** | **Left frontal and temporal lobes** | **Broca's aphasia** |
| **rTMS** | **PA8** | **Left frontal, parietal, occipital and temporal lobes** | **Transcortical motor aphasia** |
| **rTMS** | **PA9** | **Left frontal, parietal and temporal lobes** | **Broca's aphasia** |
| **rTMS** | **PA10** | **Left temporal lobe** | **Anomic aphasia** |
| **rTMS** | **PA11** | **Left temporal, parietal lobe and basal ganglia** | **Global aphasia** |
| **rTMS** | **PA12** | **Left parietal, temporal lobe and insula** | **Broca's aphasia** |
| **rTMS** | **PA13** | **Left frontal, parietal, temporal lobe and basal ganglia** | **Wernicke's aphasia** |
| **rTMS** | **PA14** | **Left temporal lobe and insula** | **Anomic aphasia** |
| **Sham** | **PA1** | **Left basal ganglia** | **Broca's aphasia** |
| **Sham** | **PA2** | **Left frontal, temporal, parietal lobe and basal ganglia** | **Transcortical motor aphasia** |
| **Sham** | **PA3** | **Left frontal lobe** | **Broca's aphasia** |
| **Sham** | **PA4** | **Left frontal and parietal lobes** | **Broca's aphasia** |
| **Sham** | **PA5** | **Left temporal and parietal lobes** | **Transcortical motor aphasia** |
| **Sham** | **PA6** | **Basal ganglia** | **Conduction aphasia** |
| **Sham** | **PA7** | **Left frontal, parietal and temporal lobes** | **Global aphasia** |
| **Sham** | **PA8** | **Left frontal lobe and thalamus** | **Anomic aphasia** |
| **Sham** | **PA9** | **Left frontal lobe and corona radiata** | **Broca's aphasia** |
| **Sham** | **PA10** | **Left frontal lobe and basal ganglia** | **Broca's aphasia** |
| **Sham** | **PA11** | **Left frontal, temporal and parietal lobes** | **Anomic aphasia** |
| **Sham** | **PA12** | **Left parietal and temporal lobes** | **Broca's aphasia** |
| **Sham** | **PA13** | **Left temporal and parietal lobes** | **Broca's aphasia** |
