# Supplementary material for: fNIRS-Guided neuronavigated rTMS augments naming recovery in subacute post-stroke aphasia: a double-blind randomized controlled trial
Source: Front Hum Neurosci. 2026 Apr 22;20:1810169. doi: 10.3389/fnhum.2026.1810169 (PMC13144099; doi:10.3389/fnhum.2026.1810169)
Supplement: Supplementary file 2 [file Table_2.DOCX]

**Supplementary Table 2. The anatomical distribution and coordinates of the targets across the 14 patients in the rTMS group**

| **Patient** | **Montreal Neurological Institute coordinates**  **(X/Y/Z)** | **Cortical region** | **Brodmann areas** |
| --- | --- | --- | --- |
| **PA1** | **-54/39/25** | **L pars triangularis Broca's area** | **45** |
| **PA2** | **-52/32/27** | **L pars triangularis Broca's area** | **45** |
| **PA3** | **-47/52/-3** | **L Dorsolateral prefrontal cortex** | **46** |
| **PA4** | **-53/-46/57** | **L Supramarginal gyrus part of Wernicke's area** | **40** |
| **PA5** | **-52/31/28** | **L pars triangularis Broca's area** | **45** |
| **PA6** | **-70/-34/7** | **L Superior Temporal Gyrus** | **22** |
| **PA7** | **-42/48/28** | **L Dorsolateral prefrontal cortex** | **46** |
| **PA8** | **-52/40/12** | **L pars triangularis Broca's area** | **45** |
| **PA9** | **-70/-22/-1** | **L Middle Temporal gyrus** | **21** |
| **PA10** | **-37/30/49** | **L Dorsolateral prefrontal cortex** | **9** |
| **PA11** | **-43/46/27** | **L pars triangularis Broca's area** | **45** |
| **PA12** | **-53/42/2** | **L pars triangularis Broca's area** | **45** |
| **PA13** | **-54/-60/50** | **L Angular gyrus, part of Wernicke's area** | **39** |
| **PA14** | **-56/20/29** | **L pars opercularis, part of Broca's area** | **44** |
